# Centre-of-Gravity Fixations in Visual Search: When Looking at Nothing Helps to Find Something

**DOI:** 10.1155/2014/237812

**Published:** 2014-06-03

**Authors:** Dustin Venini, Roger W. Remington, Gernot Horstmann, Stefanie I. Becker

**Affiliations:** ^1^The University of Queensland, Brisbane, Australia; ^2^School of Psychology, The University of Queensland, McElwain Building, Brisbane, QLD 4072, Australia; ^3^Centre for Interdisciplinary Research, Bielefeld University, 33602 Bielefeld, Germany; ^4^The University of Bielefeld, Bielefeld, Germany

## Abstract

In visual search, some fixations are made between stimuli on empty regions, commonly referred to as “centre-of-gravity” fixations (henceforth: COG fixations). Previous studies have shown that observers with task expertise show more COG fixations than novices. This led to the view that COG fixations reflect simultaneous encoding of multiple stimuli, allowing more efficient processing of task-related items. The present study tested whether COG fixations also aid performance in visual search tasks with unfamiliar and abstract stimuli. Moreover, to provide evidence for the multiple-item processing view, we analysed the effects of COG fixations on the number and dwell times of stimulus fixations. The results showed that (1) search efficiency increased with increasing COG fixations even in search for unfamiliar stimuli and in the absence of special higher-order skills, (2) COG fixations reliably reduced the number of stimulus fixations and their dwell times, indicating processing of multiple distractors, and (3) the proportion of COG fixations was dynamically adapted to potential information gain of COG locations. A second experiment showed that COG fixations are diminished when stimulus positions unpredictably vary across trials. Together, the results support the multiple-item processing view, which has important implications for current theories of visual search.

## 1. Introduction

Researchers have long been intrigued by the fact that our rich and stable visual world is created from snapshot impressions gained during short fixations on different regions of the visual field. A common assumption is that we search the visual world through a series of fixations on objects or informative portions of objects. Interestingly, we also frequently make fixations between objects into empty regions of visual space. In previous studies, such fixations have been labelled* centre-of-gravity fixations, averaging saccades,* or the* global *effect [1–4]. Here, we will use the abbreviation* COG fixations *to refer to such fixations.

COG fixations were first discovered in a saccade task, where observers were instructed to make a fast eye movement to a predefined target stimulus [5]. An irrelevant distractor in the vicinity of the target frequently led to the eyes landing at an intermediate location between the two stimuli [1, 5]. COG fixations occur only when the target and distractor were located quite near to one another, where the distance between the objects creates an angle of less than 30 degrees [2, 6–8]. Apparently, eye movements are occasionally targeted to the average of two stimulus positions rather than the precise target or distractor position.

Averaging of stimulus positions was initially attributed to the poor spatial resolution of an early saccade targeting mechanism that relies on distributed coding in a population of cells with large and overlapping receptive fields in the superior colliculus [2, 9–12]. In contrast to this bottom-up explanation, it was later noticed that fixating between stimuli may confer advantages in visual search or detection tasks, and hence, COG fixations could in part be strategic [13–15]. In particular, difficult search tasks with large numbers of distractors can potentially profit from COG fixations, as they may allow simultaneous processing of multiple stimuli during a single fixation.

In line with this contention, some studies found that COG fixations benefit task performance. For instance, investigating eye movement behaviour of chess experts and intermediate players in a check detection task, Reingold, Charness, Pomplun, and Stampe [16] found a large portion of eye fixations on squares not occupied by any chess pieces. Interestingly, experts showed significantly more COG fixations (~60%) than intermediate players (~40%) and clearly outperformed intermediate players. Reingold and colleagues [16] argued that COG fixations are a hallmark of more efficient perceptual encoding of chess configurations and thus can be viewed as a correlate for the ability to select and process multiple items in parallel.

Although the findings render it very probable that COG fixations can indeed be regarded as a correlate for more efficient perceptual encoding, the evidence for that claim is still quite indirect: firstly, because the comparisons relied on different groups of individuals that already differed in the central performance measure (experts versus novices) and second, because the effects of more efficient coding during COG fixations were not independently assessed. For instance, if COG fixations indeed allow for more efficient coding of stimulus configurations, the number of fixations on nearby stimuli and the dwell times of these stimulus fixations should be reduced. Such a result pattern would provide the most direct evidence that COG fixations enhance performance by allowing for more efficient coding of stimulus configurations.

## 2. Experiment  1

The aim of Experiment 1 was to test whether COG fixations can indeed serve as a correlate for more efficient encoding of visual stimuli, when participants do not differ in higher-order skills, and the task requires processing of abstract and unfamiliar stimuli. Observers in Experiment 1 had to search for a ring among 15 Landolt C distractors and to report with a button press whether the target ring was present or absent (50%). To test whether a higher proportion of COG fixations indeed reliably predict higher levels of visual search performance, we first correlated the observers' individual proportion of COG fixations with their performance in the visual search task (as measured by the mean response times; RTs). Secondly, to obtain independent evidence of the hypothesis that COG fixations allow processing of multiple stimuli in the vicinity, we assessed the effects of COG fixations on stimulus fixations in the same cluster of stimuli.

**Figure 1:**
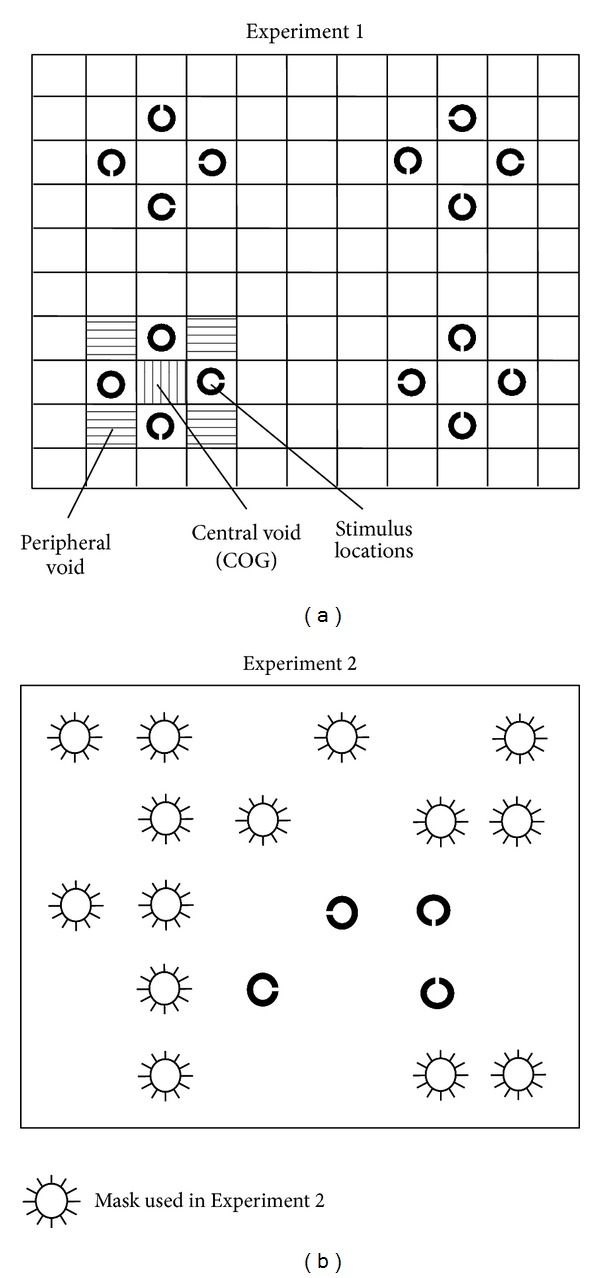
Example of the search displays used in Experiments 1 and 2. The grid (a) shows how the search display was partitioned for data analysis and was not visible in the experiment. In Experiment 1 (a), each cluster was partitioned into 4 stimulus regions (Landolt Cs), a central void region (marked by vertical lines), and 4 peripheral void regions (marked by horizontal lines). In Experiment 2 (b), a regular grid of same-sized squares was laid over the search display that contained 18 stimuli that were randomly distributed on 36 possible positions, resulting in equal numbers of stimulus regions and void regions.

Stimulus displays consisted of 4 clusters of 4 stimuli that were arranged in a diamond configuration, one in each quadrant of the search display (see Figure 1 for an example of the stimulus display). Within each diamond cluster, there were 4 stimulus locations and 5 empty locations called* void* locations: the* central void location *was also called the “COG” location, and fixations into this location were labelled COG fixations. The four void locations at the corners of the configuration were called* peripheral void locations*, and the empty space between different clusters was labelled* outside locations *(as they were outside of the clusters).

If COG fixations indeed aid search by allowing for simultaneous processing of multiple items, then COG fixations should lead to a reduction of the number of stimulus fixations and/or their dwell times within the same cluster. Moreover, these reductions should be more pronounced for COG fixations than fixations into one of the peripheral void locations. This holds because the central location offers more optimal viewing conditions of the stimuli within the cluster, given that the resolution of stimuli dramatically decreases with increases of their distance from the fovea [17].

As an additional manipulation check, we compared the effects of COG fixations between a* narrow spacing condition *versus a* wide spacing condition*, in which the stimuli were located near versus far from the centre of the diamond. By the same logic (of decreasing resolution with an increase of distance), the wide spacing condition should show less of a benefit from COG fixations than the narrow spacing condition.

In addition, comparing wide versus narrow spacing conditions, and COG fixations with peripheral void fixations, also allowed a first assessment whether and to what extent COG fixations may be under strategic control. If the COG fixations are intentionally made to process multiple stimuli simultaneously, then we would expect less COG fixations in the wide spacing condition than in the narrow spacing condition and less fixations into peripheral void locations than the central void location. However, a corresponding result may still be consistent with a bottom-up account, which attributes COG fixations to automatic averaging of stimulus positions, because averaging is more likely to occur with a narrow spacing of stimuli [1–3].

The question of whether COG fixations are strategic was tested more directly, by including an additional time-limited, narrow spacing condition. In this condition, all search stimuli disappeared after 1,500 ms, rendering it impossible to select and process all 16 search stimuli individually in a serial manner. Hence, if COG fixations indeed promote simultaneous processing of multiple items, and the visual system can exploit this fact in the optimization of search performance, then we should observe more COG fixations in this condition than the standard (unlimited) narrow spacing condition.

### 2.1. Method

#### 2.1.1. Participants

20 naïve volunteers (15 females; mean age 22.9) with normal or corrected-to-normal vision took part in the experiments and were paid $10 in compensation.

#### 2.1.2. Apparatus

The stimuli were presented on a 17′′ flat-screen colour monitor. The experiment was controlled by the software “Presentation” (Neurobehavioral Systems) that was run on an Intel Duo 2 CPU 2.4 GHz computer. Eye movements were measured with a video-based eye tracker (EyeLink 1000, SR Research, Ontario, Canada) at 500 Hz. Participants were seated in a normally lit room, with their head resting against the eye tracker's chin rest and forehead support, and viewed the screen from a distance of 62 cm. For registration of manual responses, a standard USB keyboard was used.

#### 2.1.3. Stimuli

Stimulus displays consisted of a regular array of search stimuli that either consisted of 16 black Landolt C stimuli (target absent trials) or 15 Landolt C stimuli and a target ring (diameter: 0.55°; line width: 0.09°; target present trials). All stimuli were presented against a white background and the Landolt Cs could have the gap oriented upwards, downwards, or to the right or left. The search stimuli were arranged in 4 diamond configurations of 4 × 4 stimuli, so that, within each cluster, there were a central region and 4 peripheral regions that were devoid of search stimuli (see Figure 1(a) for an example).

In the wide spacing condition, stimuli within a cluster had a distance of 7.85° (centre-to-centre), whereas in the narrow spacing condition, the distance between stimuli was 3.7° (centre-to-centre).

#### 2.1.4. Design

The experiment consisted of 3 blocked conditions of a wide spacing condition, a narrow spacing condition, and a time-limited (narrow-spacing) condition whose order was varied randomly between participants. Each block contained 180 trials, and the position of the search target and target presence was varied randomly within each block. In the time-limited condition, presentation of the narrowly spaced search display was limited to 1,500 ms, so that observers could only make 4-5 fixations before the search display disappeared. In the other conditions, the search display remained visible until response. Observers were instructed to respond as fast as possible without making any errors and were given no specific instructions about their eye movements.

#### 2.1.5. Procedure 

Prior to each block, participants were given written instructions about the next block. Each trial started with the presentation of a small black fixation cross (0.23° × 0.23°). Participants had to fixate on the centre of the cross, and the search display was presented when the gaze was within 1.3° of the centre of the fixation cross, for at least 500 ms (within a time window of 2,000 ms). Otherwise, participants were calibrated anew (9-point calibration) and the next trial started again with the fixation control.

### 2.2. Analysis

For the analysis of fixations, each cluster was divided into regions of 9 equally sized squares, consisting of four stimulus regions, one central void region, and four peripheral void regions. The remaining areas above, below, and between individual clusters were defined as “outside” regions (see Figure 1). The* a priori* probabilities of selecting a stimulus versus the central void region versus a peripheral void region were 4 : 1 : 4 across all conditions (whereby the probability of selecting an outside region was much higher in the narrow spacing condition than in the wide spacing condition).

### 2.3. Results

#### 2.3.1. Data

Trials with RTs <200 ms and >7,000 ms and wrong responses were excluded from all analyses, as were all trials where no fixations were recorded (<1%). Excluding all trials with manual errors removed an additional 17.5% of trials (most of which because of poor performance in the time-limited condition). On average, data were based on 74 correct trials per cell (range: 29 to 100 data per cell). For the reported analyses, successive fixations in the same regions were counted as a single fixation; analyses counting them as two fixations yielded very similar results.

**Figure 2:**
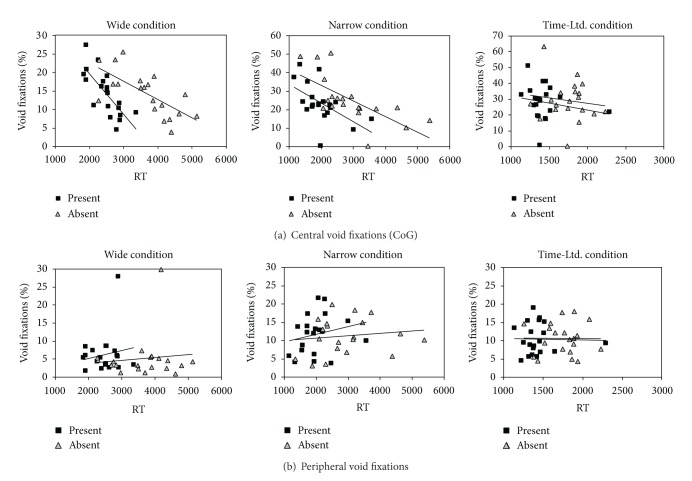
Effects of CoG fixations on search performance (RT), Experiment 1. Mean search times as a function of the proportion of void fixations in Experiment 1, depicted separately for each blocked condition (wide, narrow, and time-limited). Larger proportions of central void fixations were associated with shorter RT, both on target absent trials (black squares) and target present trials (grey triangles). Error bars depict ± 1 SEM and may be smaller than the plotting symbol.

*(I) Effect of Void Fixations on Search Efficiency*. In a first analysis, we analysed mean RT as a function of the proportion of COG fixations and fixations made into a peripheral void region (see Figure 2). First, for COG fixations, the linear regression confirmed that RT systematically decreased with a higher proportion of COG fixations, both in the wide spacing condition (present: *F*(1,19) = 23.2, *P* < .001, *R* ^2^ = .56; absent: *F*(1,19) = 16.5, *P* = .001, *R* ^2^ = .48) and in the narrow spacing condition (present: *F*(1,19) = 7.8, *P* = .012, *R* ^2^ = .30; absent: *F*(1,19) = 15.0, *P* = .001, *R* ^2^ = .45). In the time-limited condition, the same trends remained nonsignificant (all *F*s < 1), presumably because the limited presentation duration reduced the variance of the data (see Figure 2(a)).

The same analysis computed over the fixations into the peripheral void region did not show any significant effects on search efficiency (all *F*s < 1). These results demonstrate that COG fixations also benefit search performance in search among abstract and unfamiliar stimulus materials and in the absence of specific skills or expertise. Moreover, the finding that search was facilitated only by COG fixations and not peripheral void fixations is in line with the view that search benefits of void fixations depend on the information gain at the fixated location (see Figure 2(b)).

*(II) Effect of Void Fixations on Other Fixations in a Stimulus Cluster.* To test whether improved search performance with COG fixations can be attributed to the fact that multiple items were processed during the COG fixation, we next analysed whether and to what extent COG fixations reduced the mean number of fixations and/or the mean dwell times of fixations into the cluster. For this cluster-based analysis, the time-limited condition and target present trials were excluded because they lacked sufficient observations.

**Figure 3:**
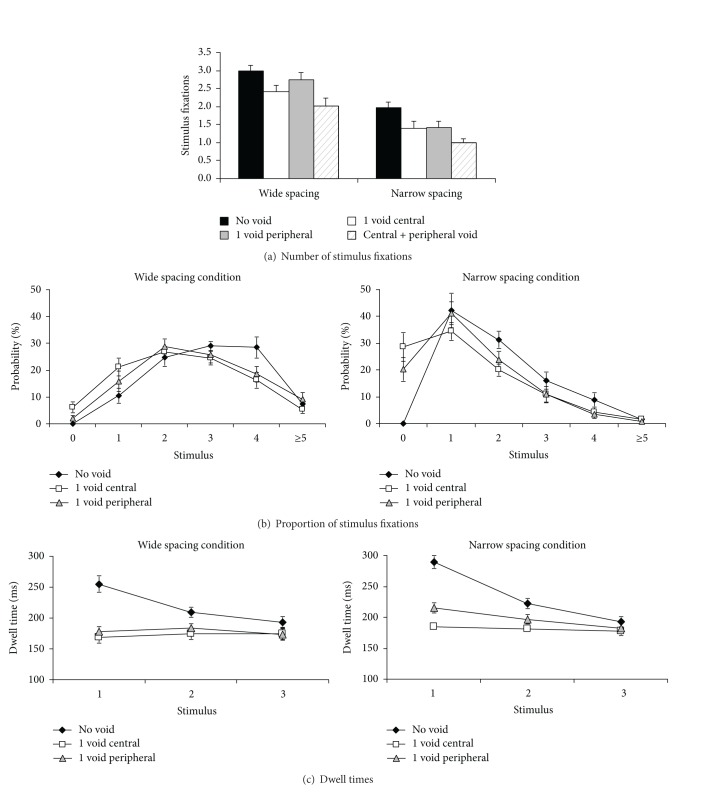
Effects of void fixations on stimulus fixations within a cluster. (a) The mean number of stimulus fixations within a cluster, depicted separately for instances in which there were no void fixations, one central or peripheral void fixation, and both central and peripheral void fixations into the cluster. Error bars depict ± 1 SEM and may be smaller than the plotting symbol. (b) The mean proportion of stimulus fixations within a cluster, depicted separately for instances in which 0, 1, 2, 3, 4, 5, or more stimulus fixations were made into a cluster. Results are depicted separately for the wide and narrow spacing conditions. (c) The mean dwell times of stimulus fixations within a given cluster, when observers had made 1, 2, or 3 stimulus fixations into a cluster, depicted separately for instances in which no void fixations, a central void fixation, or peripheral void fixations have been made into the cluster.

*(a) Number of Fixations. *The results of the first analysis confirmed that COG fixations significantly reduced the mean number of fixations into the same cluster.  Figure 3(a) depicts the mean number of fixations observed with no void fixations versus peripheral or central void fixations, or both. A 2 × 4 ANOVA comprising the variables spacing condition (narrow, wide) and fixated item (stimulus, central void, peripheral void, outside region) showed that there were more stimulus fixations in the wide condition than the narrow condition (*F*(1,18) = 35.8, *P* < .001, *η*_*p*_^2^ = .66) and more stimulus fixations when no void fixations had been made (*F*(3,54) = 53.9, *P* < .001, *η*_*p*_^2^ = .75; interaction: *F*(3,54) = 2.5, *P* = .093). Stimulus fixations were most strongly reduced when both central and peripheral void fixations had occurred, to a lesser degree with a single central void fixation and to an even lesser degree with a single peripheral void fixation (see Figure 3(a)). Two-tailed *t*-tests showed that these differences were all significant (wide spacing: all *t*s > 2.8, *P*s < .011; narrow spacing: all *t*s > 3.7, *P*s < .003), except that the effects of peripheral and central void fications did not differ in the narrow spacing condition (*t* < 1).

Figure 3(b) depicts the probability distribution of selecting 0, 1, 2, 3, 4, 5, or more stimuli within a cluster when there were no void fixations or a central or peripheral void fixation into the cluster. As shown in the figure, central void fixations mainly differed from peripheral void fixations by increasing the probability that observers made no stimulus fixations into the cluster (wide condition: *t*(19) = 2.5, *P* = .024; narrow condition: *t*(19) = 2.4, *P* = .029). In the wide condition, COG fixations also reduced the probability of making a single stimulus fixation into the cluster more than peripheral void fixations, although this difference just failed to reach significance (*t*(19) = 2.0, *P* = .059).

*(b) Dwell Times. *The same analyses were conducted to assess whether COG fixations would reduce the dwell times of stimulus fixations into the same cluster.  Figure 3(c) depicts the mean dwell times of stimulus fixations, separately for instances in which one, two, or three stimulus fixations were made into a cluster, and as a function of whether there were no void fixations or a central or peripheral void fixation. As shown in the figure, COG fixations systematically reduced the dwell times of stimulus fixations within the same cluster. A 3 × 3 ANOVA comprising the factors number of fixations (1–3) and void fixations (no void fixation versus one central void condition versus one peripheral void fixation) computed over the data of the wide spacing condition showed significant main effects of both variables (number of fixations: *F*(2,20) = 37.0, *P* < .001, *η*_*p*_^2^ = .79; void fixations: *F*(2,20) = 4.95, *P* = .039, *η*_*p*_^2^ = .33) as well as a significant interaction between the variables, *F*(4,40) = 11.0, *P* < .001, *η*_*p*_^2^ = .52. The same results were obtained in the narrow spacing condition (number of fixations: *F*(2,20) = 34.7, *P* < .001, *η*_*p*_^2^ = .72; void fixations: *F*(2,20) = 21.5, *P* < .001, *η*_*p*_^2^ = .62; interaction: *F*(4,40) = 31.8, *P* < .001, *η*_*p*_^2^ = .71). As shown in Figure 3(c), void fixations significantly reduced the dwell times of stimulus fixations, whereby this effect was particularly strong when only one stimulus fixation had been made into the same cluster. Two-tailed *t*-tests showed that in the wide spacing condition, both central and peripheral void fixations significantly shortened dwell times when participants made 1, 2, or 3 stimulus fixations into the cluster (all *t*s > 4.6, *P*s < .002); whereby the effects of COG fixations did not differ significantly from the effect of peripheral void fixations in the wide spacing condition. In the narrow spacing condition, a COG fixation significantly reduced the dwell times of stimulus fixations across all conditions (all *t*s > 3.1, *P*s < .008), and peripheral void fixations significantly reduced dwell times when observers made only 1 or 2 stimulus fixations into a cluster (*t*s > 5.0, *P*s < .001) but not when observers made 3 stimulus fixations (*t* < 1). In the narrow spacing condition, COG fixations reduced dwell times of stimulus fixations to a greater extent than peripheral void fixations when observers made one or two stimulus fixations (*t*s > 2.3, *P*s < .033), but not when 3 stimulus fixations were made into the cluster, *t* < 1.

The results listed above clearly show that an additional fixation into void regions reduced the number of stimulus fixations and the dwell times of these stimulus fixations in the same cluster, testifying to the fact that fixations into empty regions aided stimulus processing. An additional analysis was conducted to test whether dwell times of stimulus fixations are more strongly reduced by void fixations than stimulus fixations. To that aim we compared dwell times between clusters with two fixations in total (two stimulus fixations, or one stimulus fixation and one void fixation). The results showed that dwell times were shorter when a void fixation had occurred than when both fixations had been stimulus fixations. In the wide spacing condition, both central and peripheral void fixations resulted in significantly shorter dwell times than a stimulus fixation, *t*s > 2.5, *P*s < .026. In the narrow condition, only central void fixations shortened dwell times significantly more than stimulus fixations, *t*(17) = 5.2, *P* < .001, whereas peripheral void fixations did not shorten dwell times more than stimulus fixations, *t* < 1. Taken together, the findings show that void fixations affect stimulus processing more strongly than stimulus fixations.

*(III) Determinants of COG Fixations and Peripheral Void Fixations.* As mentioned above, the results so far support the view that the visual system can profit from fixations on empty locations, with the gain related to the possible information gain at a location. Can the visual system in addition strategically use void fixations to maximise such gains? If this is the case, then we would expect more COG fixations on the central void region than the peripheral void regions and more COG fixations in the narrow spacing condition than the wide spacing condition. This holds because, in these conditions, COG fixations have a higher potential information gain. However, corresponding results may still be consistent with a bottom-up account of COG fixations because of the underlying differences in the stimulus displays (wide versus narrow spacing) or the relative positions (central versus peripheral void regions exerting different bottom-up effects on eye movements). Hence, the most decisive evidence for a strategic account of COG fixations would be an increase of COG fixations in the time-limited condition compared with the (unrestricted) narrow spacing condition, which had the same stimulus displays.

**Figure 4:**
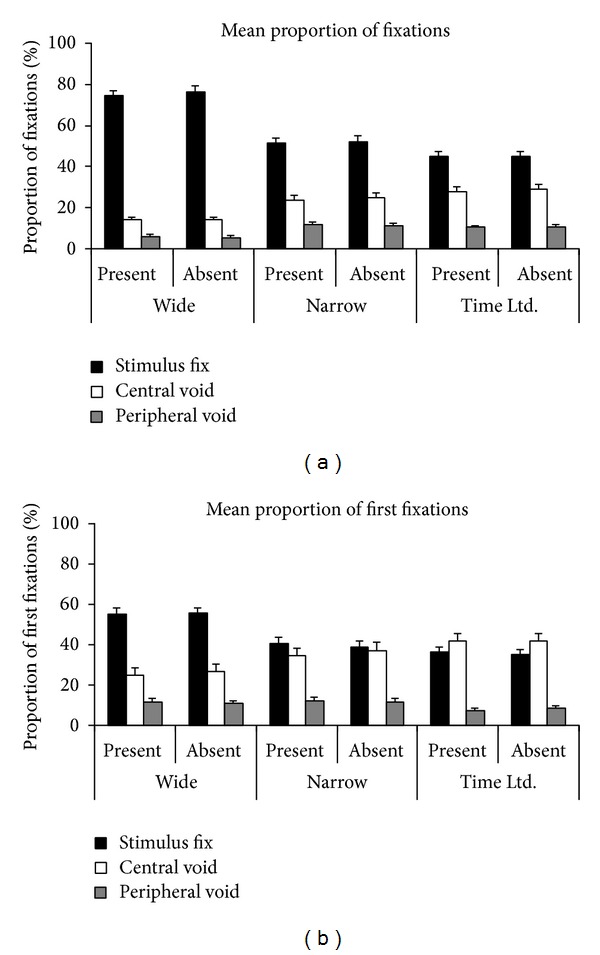
Determinants of CoG fixations and peripheral void fixations. The mean proportion of fixations (a) and mean proportion of first fixations (b) within a trial, depicted separately for the different conditions and different types of fixations (stimulus fixation versus central void fixation versus peripheral void fixation). Error bars depict ± 1 SEM and may not be visible when the SEM is very small.

Figure 4 depicts the results of the three analyses. In line with the predictions of a strategic account, the central void region was more frequently selected in the time-limited condition than in the unrestricted narrow spacing condition, on both target present trials, *t*(19) = 3.7, *P* = .002, and target absent trials, *t*(19) = 2.4, *P* = .025 (see Figure 4(a)). These results indicate that the proportion of COG fixations was indeed strategically adapted to the task demands, indicating that we have at least partial top-down control over COG fixations.

Comparing the proportion of COG fixations across different regions and conditions showed that the central void region was selected more frequently than the peripheral void regions, on target present and absent trials and across all spacing conditions (all *t*s > 3.6; all *P*s < .003). Moreover, the proportion of COG fixations was higher in the narrow spacing conditions than in the wide spacing condition, on both target present and target absent trials (all *t*s > 5.4; *P*s < .001). These results demonstrate that the proportion of COG fixations was flexibly adapted to the task demands and the projected information gains.

An additional analysis was performed over the first fixations within a trial, to check whether the observed effects were present at an early stage of visual processing or whether they developed later during search (see Figure 4(b)). Contrary to the latter contention, selection of the central void region was disproportionately high in the first fixation within a trial—significantly higher than in the average proportion of fixations (*F*(1,19) = 81.1, *P* < .001, *η*_*p*_^2^ = .81), and this was true for all spacing conditions and for target present and absent trials (all *t*s > 5.3, *P*s < .001). In line with the earlier results, a higher proportion of first fixations was made to the COG region rather than a peripheral void region, across all conditions (all *t*s > 2.8, *P*s < .012); the narrow and time-limited conditions showed significantly higher proportions of first COG fixations than the wide spacing condition (all *t*s > 2.2, *P*s < .036), and there were significantly or marginally significantly more first COG fixations in the time-limited condition than in the narrow spacing condition (present trials: *t*(19) = 3.5, *P* = .002; absent trials: *t*(19) = 1.9, *P* = .068). Taken together, these results demonstrate that strategies to adapt COG fixations to the information gain of a condition or location were present at an early stage.

### 2.4. Discussion

The results of Experiment 1 support the view that COG fixations can be regarded as a correlate for multiple-item processing: first, COG fixations significantly benefited search performance, and second, COG fixations reliably reduced the number of stimulus fixations and their dwell times. Of note, fixations on the peripheral void regions did not enhance search efficiency and reduced stimulus fixations and their dwell times to a lesser degree than true COG fixations. These findings may constitute the best available evidence that multiple items were processed during a COG fixation.

In addition, the results demonstrated that COG fixations are to some degree under top-down control, as reflected by the results of the time-limited condition: when observers were forced to search quickly through the stimuli, the proportion of COG fixations increased significantly. These results are not predicted by a bottom-up account of COG fixations and indicate that COG fixations were strategically adapted to the task demands.

The results of the manipulation checks further corroborated these findings: more fixations were made into the highly informative central void region than the lesser informative, peripheral void regions, and more COG fixations were made in the narrow spacing condition than in the wide spacing condition. These results are consistent with the view that fixations into empty regions depend on the information content in a particular region, with their landing position and frequency being strategically adapted to optimise stimulus processing [16, 18, 19].

A possible complication for this interpretation is that COG fixations were frequently made at the beginning of the trial. Given that trials with short RT will have fewer fixations overall, it is possible that trials with short RT had a higher proportion of COG fixations simply because trials with short RT have fewer fixations overall. To address this potential problem, we inspected entire trials according to whether they showed a COG fixation or not and computed the proportion of trials that had one or more COG fixations (by dividing the number of trials with COG fixation by the number of all trials). Analyses based on the proportion of trials with COG fixations are biased against finding facilitatory effects of COG fixations, because the probability of a COG fixation (or any kind of fixation) is lower when trial times are short (i.e., with short RT). This holds because fixations take time and, therefore, are usually positively correlated with RT.

As shown in Table 1, RT indeed correlated positively with stimulus fixations and peripheral void fixations. However, the reverse effect of a negative correlation was evident for central void (COG) fixations. That is, across all conditions, participants with a higher proportion of trials with COG fixations showed faster RT (see Table 1). The results of the RT regressions showed that this negative correlation was significant only on wide present trials, whereas the positive correlation between stimulus fixations and RT was always significant, and the positive correlation between peripheral void fixations and RT was significant for the narrow condition. This result was expected, considering the bias against finding a negative correlation.

Note that COG and stimulus fixations were collated independently of each other, thus allowing for the conclusion that a higher proportion of trials with COG fixations (and with or without stimulus fixations) result in shorter RT whereas a higher proportion of trials with stimulus fixations (and with or without COG fixations) result in longer RT. Taken together, the results support the notion that COG fixations benefit search—presumably, because a higher proportion of COG fixations (indirectly) indexes an observer's capacity for processing multiple items in parallel.

We also compared RT between trials in which the first fixation was made on a central or peripheral void region or on a stimulus. Three subjects had to be excluded from these analyses because of missing values. The wide condition showed shorter RT on present trials when the first fixation had been a COG fixation (2,284 ms) than when it had been a stimulus fixation (2,500 ms; *t*(16) = 3.2, *P* = .006) and shorter RT on absent trials when the first fixation had been a COG fixation (3,586 ms) than when it had been a stimulus fixation (3,691 ms; *t*(16) = 2.1, *P* = .053) or a peripheral void fixation (3,789 ms, *t*(16) = 2.4, *P* = .029). The narrow condition showed the same trends which, however, remained nonsignificant.

Despite the clear evidence that COG fixations benefit search, it could still be doubted that COG fixations are affected by top-down strategies. More COG fixations in the time-limited condition could also be due to saccades being elicited earlier, with the result that saccade endpoint positions were based on early sensory signals with a poor resolution that simply averaged over positions [2, 6, 7, 9, 11]. In this case, more COG fixations in the time-limited condition would be simply due to bottom-up limitations in the visual system and the fact that the time restrictions left no time to provide high-resolution information about the object position early on in saccade planning.

**Figure 5:**
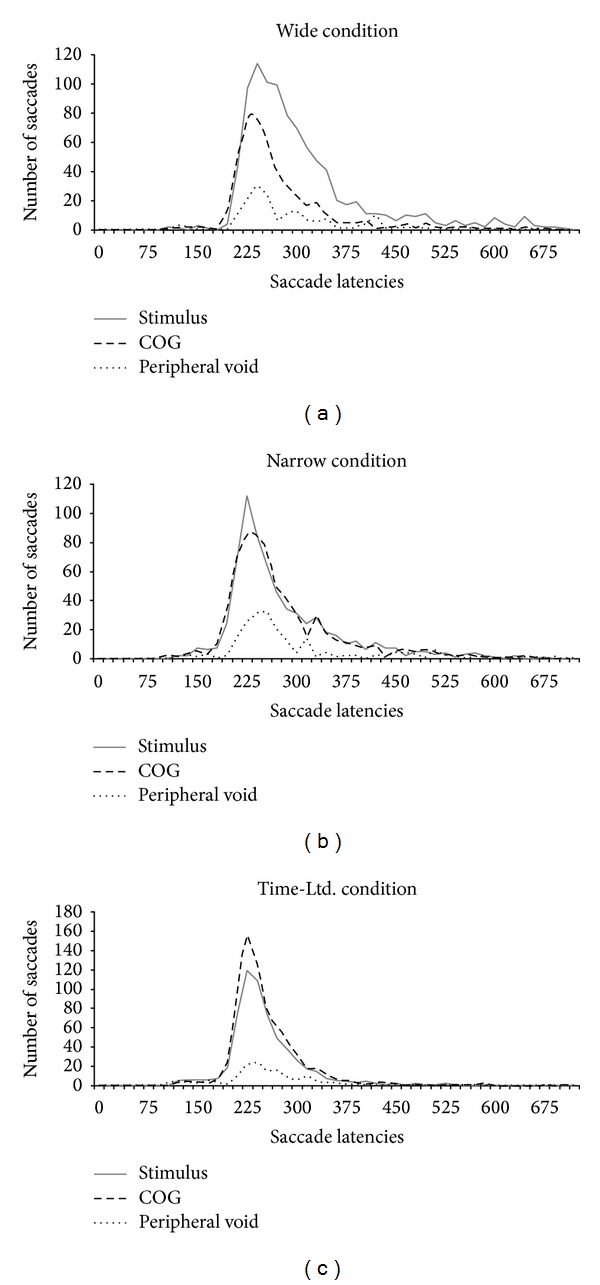
Fixations as a function of saccade latency. Number of fixations on a stimulus, central void region (COG), or peripheral void region as a function of saccadic latency, depicted separately for the wide and narrow spacing conditions and the time-limited narrow spacing condition.

We tested this bottom-up explanation by inspecting whether the first saccades within a trial directed at a (central or peripheral) void region indeed started earlier (i.e., had shorter saccade latencies) than saccades directed at a stimulus. To ensure that the first saccades were not unduly influenced by the target, we limited the analyses to target absent trials and collapsed the data over correct and incorrect trials to yield sufficient observations. Figures 5(a)–5(c) depict the number of trials in which the latencies of the first saccades was short versus long (0–700 ms saccade latency, in bins of 15 ms), separately for stimulus, COG, and peripheral void fixations. As shown in Figure 5, COG fixations were not made visibly earlier than stimulus fixations. This rules out that COG fixations in the time-limited condition were due to bottom-up limitations that increased saccades into the centre of gravity because of poorly resolved sensory signals [2, 9]. Instead, in all conditions (wide, narrow, and time-limited), the earliest saccades directed either to a stimulus or the centre of gravity were elicited around 170–195 ms, independently of whether the conditions had time limitations. These results indicate that the higher proportion of COG fixations in the time-limited condition were due to a strategic adaptation to the task demands, with the COG location being targeted more frequently because it optimized the information gain.

Of note, Experiment 1 showed an unusually high number of COG fixations: previous visual search studies usually reported much lower proportions of COG fixations that were well below 20% [11, 12, 14]. In the previous literature, higher proportions of COG fixations seemed to be limited to studies using meaningful stimuli such as chess configurations or words that were embedded in a highly structured context [14, 20–23]. The present finding of high proportions of COG fixations especially in the first fixations (~50%) demonstrates that high proportions of COG fixations can be observed with unfamiliar and abstract stimulus materials. Hence, the present findings show that expertise or higher-order cognitive skills such as reading or chess expertise are not necessary for observers to select COG locations.

It should be noted, however, that the stimuli in the present study were arranged in highly structured clusters. Experiment 2 was designed to test whether the same results could be obtained in unstructured displays, when the stimulus positions were unforeseeable.

## 3. Experiment  2

Experiment 2 was designed to test whether COG fixations would still aid search and show evidence for being strategic when the search stimuli are presented in unstructured and unforeseeable configurations. To that aim, we used the same stimuli and task in Experiment 2 as in the previous experiment; however, in Experiment 2, 18 Landolt Cs were distributed randomly over 36 possible locations in the search array. This allows for an equal number of grid regions to contain either a Landolt C or an empty space (see Figure 1(b) for reference). The viewing time was limited (to 5,500 ms) to encourage participants to adopt an efficient search strategy.

As in Experiment 1, we assessed whether COG fixations facilitate search by computing a regression over the mean RTs with COG fixations as an independent variable. To test whether COG fixations observed in this task were strategic, we dynamically adapted the size of the viewable area around the observer's gaze location: all search stimuli (Landolt Cs) were initially masked with stimuli that had the same size as the search stimuli but did not convey any information about whether the location contained a target or a distractor. In different blocked conditions, a different proportion of search stimuli were unmasked within a gaze-contingent window that was continually centred at the observer's gaze, with the window unmasking either only a single stimulus (i.e., the fixated stimulus,* Z70 condition*), 2-3 stimuli (*Z150 condition*), 3-4 stimuli (*Z200 condition*), a whole quadrant (*Zoom320 condition*), or all stimuli (*free viewing condition*). If COG fixations are indeed under top-down control, the proportion of COG fixations should increase with the window size (i.e., the area of viewable search stimuli), because the possible information gain of COG fixation progressively increases with the window size. Hence, if participants can strategically use COG fixations to promote multiple-item processing, COG fixations should gradually increase along with an increase in window size. By contrast, if COG fixations are purely bottom-up, they should not be modulated by the potential information gain of between-item fixations and, thus, should not differ between the conditions because the masks did not alter the stimulus locations or their configuration.

### 3.1. Method

#### 3.1.1. Participants

Twelve new and naïve participants (nine females, mean age of 20.1) took part in Experiment 2 and were reimbursed with $10 for their participation.

#### 3.1.2. Stimuli, Design, and Procedure

The observers' task was to search for a closed target ring among distractor Landolt Cs (diameter: 0.83°) in an array of 18 stimuli that were randomly positioned on a 6 × 6 matrix (20.3°× 20.3°; distance between stimuli: 4.1°). As in Experiment 1, observers had to report with a button press whether the target was absent or present (50%).

In the free viewing condition, all stimuli were visible from the start of the trial. In the restricted viewing conditions, only stimuli (Landolt Cs and target ring) within a specific distance around the fixation were visible, whereas all other stimuli were obscured by masks. The masks were circles with thin intersecting lines at the outlines and a small dot in the centre (see Figure 1(b), bottom). In the restricted viewing conditions, the size of the gaze-contingent window varied in different blocks between 70 pixels (1.9°), 150 pixels (4.2°), 200 pixels (5.5°), and 320 pixels (8.9°). Across all conditions, the search stimuli were presented for 5,500 ms, and participants completed 55 trials in each block. All participants were familiarised with the task by first completing 20 trials of the free viewing condition. After familiarisation, all participants first worked through the free viewing condition, after which they completed 4 blocks of the restricted viewing conditions, which were presented in random order.

### 3.2. Data

In Experiment 2, RTs longer than 10 s were excluded from all analyses (<0.1%). Trials with wrong manual responses (15.1%) were also excluded. Mean values were based on 27 trials per cell (range: 19 to 36 trials).

### 3.3. Results

**Figure 6:**
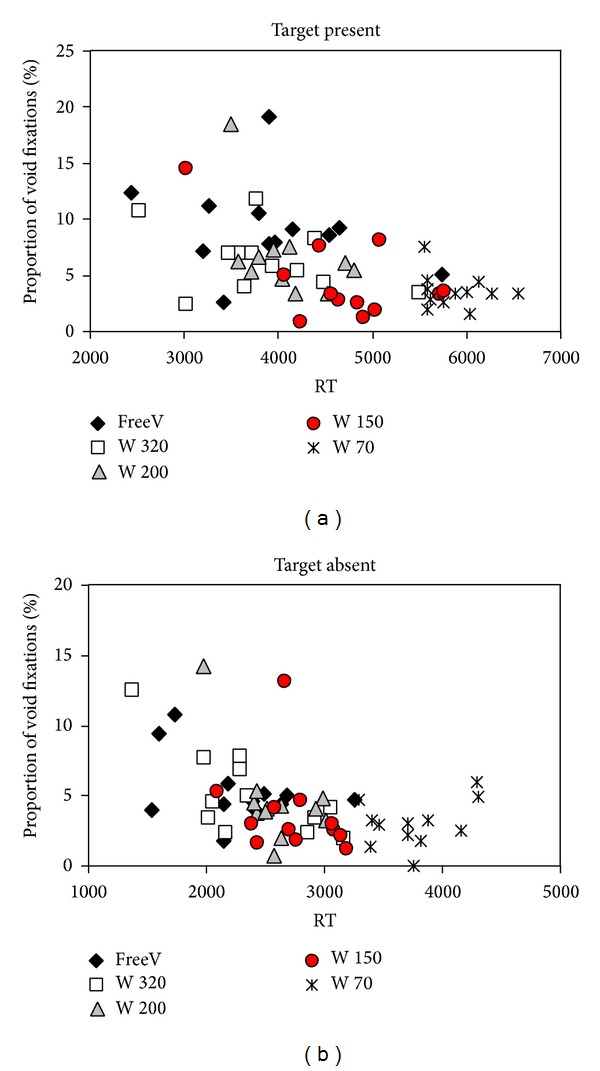
Effects of COG fixations on search performance (RT), Experiment 2. Mean individual search times as a function of the proportion of fixations into a void region, depicted separately for the free viewing condition (FreeV; black diamonds) and the 4 different restricted viewing conditions (W = window, with number specifying the size of the viewable area in pixels). As in Experiment 1, a higher proportion of void fixations is associated with shorter RT, both on target present trials (a) and target absent trials (b).

Table 2 shows the effect of window size on the mean RT, error scores, dwell times, and fixation parameters. As can be seen in Table 2, both the mean RT and the number of stimulus fixations significantly decreased as the window size increased. To assess the effect of COG fixations on search performance, we computed a linear regression over the mean RT, with window size (1–5) and proportion of COG fixations as independent variables (see Figure 6). The analysis showed that both the proportion of COG fixations and the window size significantly modulated RT (present trials: *F*(2,59) = 39.4; *P* < .001; *R* ^2^ = .58; COG fixations: *t* = 3.0; *P* = .004; window size: *t* = 7.0; *P* < .001; absent trials: *F*(2,59) = 31.8; *P* < .001; *R* ^2^ = .53; COG fixations: *t* = 2.9; *P* = .015; window size: *t* = 5.4; *P* < .001). As in Experiment 1, search was significantly faster with a higher proportion of COG fixations, indicating that COG fixations facilitated search.

Table 2 shows that the proportion of COG fixations was higher for the first fixations on a trial than averaged over all fixations within a trial (compare Proportion void fix with Proportion of 1st void). This again raises the problem that short RT may have correlated with a higher proportion of COG fixations simply because COG fixations predominantly occurred early in the trial. To ascertain that COG fixations indeed aid search performance, we additionally computed the RT regression over the individual's mean proportion of trials with COG fixations. The results showed that narrowing the window size significantly decreased the proportion of trials with COG fixations (*F*(4,44) = 3.3, *P* = .034). Moreover, a linear regression showed that a higher proportion of COG trials still led to significantly faster RT on absent trials, *F*(1,59) = 7.3, *P* = .009, *R* ^2^ = .11, whereas the same trends on target present trial remained nonsignificant, *F* < 1. These results show that COG fixations facilitate search even in unstructured displays.

### 3.4. Discussion

The results of the second experiment showed that COG fixations can also serve as a correlate for search efficiency in visual search among unstructured displays, in which stimulus positions are unpredictable. These results reinforce the view that COG fixations facilitate processing of multiple items in the periphery.

Moreover, as in Experiment 1, the frequency of COG fixations was adapted to different viewing conditions, with progressively fewer COG fixations when the viewing area was further limited and there were fewer opportunities to process multiple items during a fixation. These results indicate that COG fixations were at least to some extent under top-down control. Of note, a pure bottom-up account cannot account for the decrease of COG fixations with smaller window size, because the masks did not differ in saliency, size, or shape from the search stimuli. Hence, these effects have to be attributed to a strategic adaptation of COG fixations. In sum, the results of Experiment 2 indicate that COG fixations probably reflect strategic adaptations of eye movement behaviour that are controlled by the potential gains of COG fixations, even in unstructured visual search displays.

COG fixations were, however, much rarer in Experiment 2 than in the previous experiment, with only about 10% of all fixations being COG fixations and less than 20% of first fixations being COG fixations, even in the free viewing condition. Although the results of Experiments 1 and 2 cannot be directly compared to each other because of the differences in stimulus size and density [3], the same observation has been made in several other studies [14–16, 21]. Together, these findings suggest that structured displays may be crucial for observing high rates of COG fixations, possibly, because in structured displays, the stimulus positions are foreseeable and allow preplanning eye movements. However, this possibility certainly warrants further investigation.

## 4. General Discussion

The present study yielded several interesting results. First, the results showed that COG fixations facilitate search by reducing the number of stimulus fixations and their dwell times within a cluster of stimuli. Second, the results showed that COG fixations were strategically adapted to different conditions, in that they were (a) more frequently made to locations with a higher potential information gain (central versus peripheral void locations), (b) more frequent in conditions that allowed for better viewing of stimuli in the vicinity (narrow spacing versus wide spacing in Experiment 1, larger area of visible search stimuli in Experiment 2), and (c) more frequently made in conditions that required an efficient selection strategy (i.e., time-limited condition in Experiment 1). In particular the latter results show that COG fixations in visual search are modulated by task demands, both in unstructured and structured displays. Thus, COG fixations reflect to some extent a strategic adaptation to maximise multi-item processing.

As a caveat, the view that COG fixations are modulated by task demands does not mean that the saccade endpoints are necessarily chosen voluntarily or chosen consciously by the observer. We use the terms top-down and strategic to point to a change in performance that is caused by the task (not the stimuli) and serves to optimise performance in the task, while permitting that such optimizations can be implicit and not consciously accessible (e.g., Becker, 2007). In addition, the present results can only show that COG fixations are modulated by top-down task demands and are in that sense strategic. This leaves open the possibility that the eye movement itself is generated as a bottom-up default option and that the saccade programme is only abandoned or executed depending on whether top-down demands favour or prohibit its occurrence [9, 11]. What the results show is that COG fixations are top-down modulated and strategic in the sense that their occurrences cannot be fully explained by bottom-up mechanisms.

As mentioned above, the view that COG fixations are not purely determined by an automatic averaging mechanism but are in part strategic has been proposed before [18]. However, the evidence for this view was not complete, as critical findings were based on comparing performance between different groups (e.g., experts versus novices). Alternatively or additionally, the tasks often involved familiar stimuli that required higher-order cognitive abilities such as chess expertise or reading skills [18, 20–24], and there was no independent evidence for the view that COG fixations indeed foster multiple-item processing. The present study complements previous work by showing that COG fixations facilitate search by allowing for simultaneous processing of multiple items and that these findings generalise to observers with no specific expertise and to tasks and stimulus materials that are unfamiliar and do not require higher-order cognitive skills.

Of note, the present results leave open the possibility that COG fixations also depend to some extent on the stimulus conditions, as there were more COG fixations in the highly structured and foreseeable displays (Experiment 1) than the displays with random stimulus position (Experiment 2). In Experiment 1, stimulus clusters were always structured the same and the configuration did not change across trials. Hence, eye movements into void regions could be made either on the basis of the remembered positions of the void regions (from the last trial) or by applying the same algorithm for computing the saccade landing position across the four clusters. It is unknown whether COG fixations can be based on remembered saccade locations, and at a first glance, it may seem implausible that regions devoid of stimuli are memorised and can be used for saccade programming. However, in the present stimulus configurations, the central void region was always situated at the centre of a diamond, so that it could be targeted by encoding the global structure of the diamond and honing in on its centre. Presenting stimuli in a global structure may thus have had a dual facilitating effect of speeding on-line computations of saccade target positions and aiding memory-related processes of encoding the locations of void regions [15]. The present study cannot determine whether a higher proportion of COG fixations in Experiment 1 was due to the fact that the diamond configurations facilitated COG saccade programming or whether repeating the displays allowed executing COG saccades from memory. However, what is clear from the results is that the stimulus conditions play an important role in bringing about COG fixations, whereas the familiarity of the stimulus material and skill-related abilities are less decisive.

Moreover, the strong dependency of COG fixations on the information gain and strategic factors also questions the claims of current theories of eye movement control, that visual selection is largely determined by bottom-up saliency [25–27], or that COG fixations reflect bottom-up averaging of positions [2, 17]. Instead, the results highlight the importance of strategic factors in guiding the gaze [19].

*Implications for Theories of Attention and Eye Movement Control.* As mentioned above, the results of the present study support the view that multiple items are processed during a single fixation. This view is strongly supported by the two findings: first, the finding that observers frequently made only a single fixation into a stimulus cluster (see Figure 3(b)) and second, the finding that dwell times on stimulus fixations varied systematically with the number of stimulus fixations and void fixations into a cluster (see Figure 3(c)). These results strongly suggest that more often than not multiple items are selected and processed during a single fixation, including “normal” stimulus fixations. To some researchers, these conclusions may not be surprising: several eye movement studies have already shown that observers typically do not select each and every stimulus present in the display, which already indicates that multiple-item processing occurs at least during some fixations [28]. However, in the analysis of eye movement data, it is typically assumed that each fixation has to be attributed to a single stimulus and, correspondingly, fixations between stimuli are usually either precluded from the outset (by defining areas of interest such that there are no gaps) or they are discarded as saccade errors [29, 30]. The results of the present study clearly reject this view. As shown in Experiment 2, even when COG fixations occur only infrequently, they significantly facilitate search. Moreover, in Experiment 1, COG fixations systematically altered the pattern of dwell times of stimulus fixations in the vicinity. These results highlight the importance of considering multiple items as the “unit of selection” in eye movement studies and suggest that excluding fixations on empty locations can falsify the results. This is perhaps an especially important point to consider for the analysis of dwell times. Of note, previous studies have often reported the rather puzzling finding that dwell times are impervious to stark modifications of the display, such as variations in the display duration or the number of items [31]. This has led to the view that that dwell times are relatively fixed and are determined prior to a trial, by a global estimate of how much processing will be required during each single fixation [31]. The results of Experiment 1 suggest that dwell times instead strongly depend on the number of fixations into a stimulus cluster, with systematically decreasing dwell times with increasing stimulus fixations. Moreover, a single void fixation can eliminate these differences and lead to dwell times that are largely unaffected by the number of stimulus fixations (see Figure 3(c)). These findings demonstrate that dwell times are strongly modulated by on-line processing demands [32], albeit the processing demands of a cluster of stimuli instead of a single stimulus. Previous failures to find on-line processing effects on dwell times may be rooted in the fact that fixations were mostly analysed with respect to a single stimulus instead of taking a restricted area of stimuli into account and analysing possible interactions between fixations into the cluster.

Taken together, the results of the present study provide new insights into how our common strategy of assigning fixations to single stimuli and excluding COG fixations can falsify the results and lead to wrong conclusions. In future studies, it may be worthwhile to consider the possibility of multiple-item processing during single fixations in the analysis of eye movement data and/or create stimulus conditions that preclude effective multiple-item processing. Considerations about adequate spacing between stimuli are also important to ascertain whether and to what extent visual search may be hampered by* crowding*—that is, limitations to discriminate the target in the periphery due to interference by nearby distractors [33].

In conclusion, we found that COG fixations lead to more efficient search and lower stimulus fixations and dwell times and that COG fixations diminish when stimulus positions unpredictably vary across trials. These results offer additional support for the multiple-item processing view and demonstrate how the area between stimuli can be of strategic value in visual search.

## Figures and Tables

**Table 1:** Correlations of RT with the proportion of trials with stimulus fixations, central void fixations, and peripheral void fixations.

	Wide spacing			Narrow spacing		
	Stim.	COG	Periph.	Stim.	COG	Periph.
Present trials	97.4%	65.5%	34.4%	91.4%	76.7	55.6%
Correl. (RT)	0.491*	−0.498*	0.223	0.532*	**−0.220**	0.581*
*P* value (Regr.)	0.028	0.025	n.s.	0.016	n.s.	0.007

Absent trials	100%	85.1%	42.2%	91.5%	76.7%	74.8%
Correl. (RT)	NA	**−0.251**	0.300	0.587*	**−0.179**	0.687*
*P* value (Regr.)	NA	n.s.	n.s.	0.007	n.s.	0.007

*Note.* Mean proportion of trials with one or more stimulus fixations, one or more COG fixations, and one or more peripheral void fixations; the correlation of each of these values with the participants' mean RT, depicted separately for the wide and narrow spacing conditions and present and absent trials. Asterisks indicate significant correlations, as per linear regressions that were computed separately over the RT and the proportion of trials with stimulus fixations, COG fixations, or peripheral void fixations, respectively (exact *P* values reported below). Bold font indicates negative correlations indicative of facilitation. Stim.: stimulus fixation; COG: central void fixation; periph.: peripheral void fixation.

**Table 2:** Experiment  2: effect of window size on eye movement parameters.

	Viewing condition					Effect of window size		
	Free V	Z 320	Z 200	Z 150	Z 70	*F*	*P*	*η*_*p*_^2^
RT								
Pres.	2267**	2381**	2585**	2748**	3763	35.9	<.001	0.76
Abs.	3809**	3860**	4127**	4696**	5877	50.4	<.001	0.82
Error								
Pres.	22.9	21.4	26.2	22.7	30.4	1.6	n.s.	
Abs.	3.4	2.2	1.8	1.0	3.7	1.7	n.s.	
Number of stim. fix.								
Pres.	6.1**	6.4**	6.8**	7.1**	9.6	15.5	<.001	0.058
Abs.	14.2*	14.0*	14.8*	16.4	16.7	5.0	.014	0.31
Number of void fix.								
Pres.	0.6**	0.4	0.5	0.3	0.4	3.7	.011	0.25
Abs.	0.8**	0.7*	0.6	0.6	0.5	3.3	.018	0.23
Dwell time								
Pres.	164**	171**	171**	185**	212	45.6	<.001	0.80
Abs.	168**	173**	177**	185**	219	48.6	<.001	0.81
Prop. stim. fix.								
Pres.	90.6**	93.2**	92.7	95.0	96.2	6.5	<.001	0.37
Abs.	94.5*	94.7	95.0	96.1	96.3	2.4	.062	(0.18)
Prop. void fix.								
Pres.	9.2**	6.4**	6.9*	4.5	3.6	9.5	<.001	0.47
Abs.	5.4**	5.2*	4.6	3.7	3.0	4.9	.002	0.31
Proportion of 1st stim.								
Pres.	81.8	82.3	82.2	90.4*	81.2	1.9	n.s.	
Abs.	81.2	84.9	83.7	86.6	80.5	1.5	n.s.	
Proportion of 1st void								
Pres.	18.2	17.7	17.8	9.2*	18.8	2.0	n.s.	
Abs.	18.8	15.1	16.0	13.4	19.5	1.6	n.s.	

*P < .05, **P < .01 for the comparison to the narrowest zoom condition (Z70).
